# The Role of Chemerin in Metabolic and Cardiovascular Disease: A Literature Review of Its Physiology and Pathology from a Nutritional Perspective

**DOI:** 10.3390/nu15132878

**Published:** 2023-06-25

**Authors:** Lunbo Tan, Xifeng Lu, A. H. Jan Danser, Koen Verdonk

**Affiliations:** 1 Division of Vascular Medicine and Pharmacology, Department of Internal Medicine, Erasmus MC, 3015 CN Rotterdam, The Netherlands; l.tan@erasmusmc.nl (L.T.); a.danser@erasmusmc.nl (A.H.J.D.); 2 Clinical Research Center, The First Affiliated Hospital of Shantou University Medical College, Shantou 515041, China; xifeng.lu@outlook.com

**Keywords:** chemerin, nutrients, cardiovascular disease, metabolic disease

## Abstract

Chemerin is a novel adipokine that plays a major role in adipogenesis and lipid metabolism. It also induces inflammation and affects insulin signaling, steroidogenesis and thermogenesis. Consequently, it likely contributes to a variety of metabolic and cardiovascular diseases, including atherosclerosis, diabetes, hypertension and pre-eclampsia. This review describes its origin and receptors, as well as its role in various diseases, and subsequently summarizes how nutrition affects its levels. It concludes that vitamin A, fat, glucose and alcohol generally upregulate chemerin, while omega-3, salt and vitamin D suppress it. Dietary measures rather than drugs acting as chemerin receptor antagonists might become a novel tool to suppress chemerin effects, thereby potentially improving the aforementioned diseases. However, more detailed studies are required to fully understand chemerin regulation.

## 1. Introduction

Over the last three decades, due to the obesity epidemic, attention has shifted to achieving an improved energy balance. The underlying concept is that a healthy lifestyle and well-controlled nutrition will avoid obesity, and consequently prevent the development of metabolic syndrome and any resulting cardiovascular disease [1]. 

Chemerin is a multifunctional protein that has recently been identified as an essential player in hypertension, myocardial infarction, preterm birth, diabetes, metabolic disease and liver cirrhosis [2,3]. In the two decades since its initial discovery, more than a thousand articles have been published on chemerin [4], but none reviewed its relationship with nutrition.

This review aims to comprehensively cover the physiology and pathological roles of chemerin from a nutritional point of view, an approach based on the literature search shown in Supplemental Figure S1. The underlying assumption is that by lowering chemerin levels through dietary interventions, novel therapeutic strategies may be identified for the prevention and treatment of various cardiovascular diseases associated with obesity and metabolic syndrome. 

## 2. Chemerin and Its Receptor

### 2.1. Origin of Chemerin

**Figure 1:**
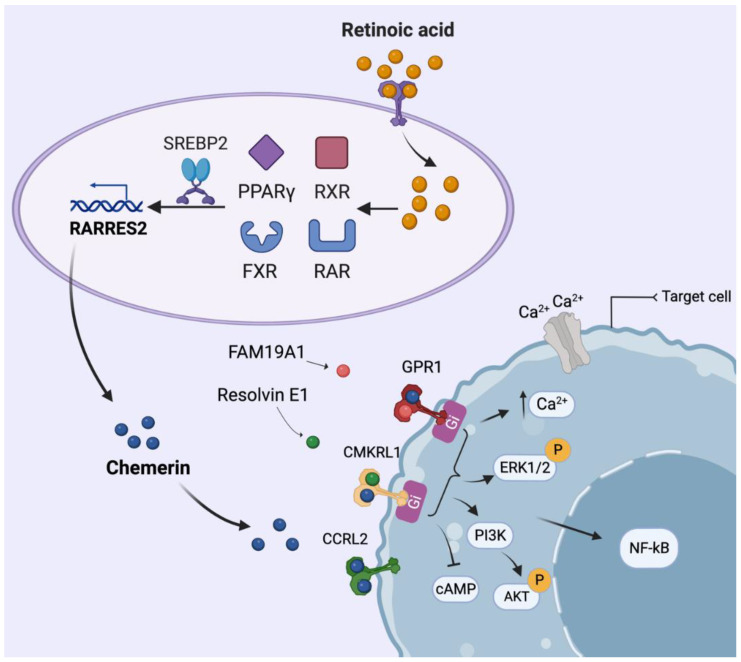
Induction of chemerin synthesis with retinoic acid, the activation of its receptors, and the resulting second messenger cascade. Not only chemerin, but also FAM19A1 and resolvin E1 target these receptors. See text for further details. RARRES2, retinoic acid receptor responder 2; FXR, farnesoid X receptor; RAR, retinoic acid receptor; RXR, retinoid X receptor; PPARγ, peroxisome proliferator-activated receptor γ; SREBP2, sterol regulatory element-binding protein 2; CMKLR1, Chemerin-like receptor 1; CCRL2, CC-motif chemokine receptor-like 2; GPR1, chemerin type 2 receptor; ERK1/2, extracellular signal-regulated kinase 1/2; NFκB, nuclear factor-κB; PI3K, phosphoinositide 3-kinase; AKT, protein kinase B.

Chemerin was first identified in 1997 [5]. It was found in psoriatic lesions, and its expression increased after topical exposure to the retinoid tazarotene, hence its first name Tazarotene-induced Gene 2 (TIG2) [5]. Given this observation, the initial focus was on retinoic acid receptors (RARs) and retinoid X receptors (RXRs), with only the former resulting in TIG2 upregulation [5]. The gene then became known as retinoic acid receptor responder 2 (RARRES2) [6]. RARRES2 was believed to be a soluble ligand for a surface receptor involved in antiproliferative effects [7]. In 2003, the protein sequence of RARRES2 was unraveled, and it received the name chemerin, while simultaneously the G protein-coupled orphan receptor ChemR23 was confirmed to be its receptor [8]. Interestingly, two nuclear receptors heterodimerizing with RXR [9,10] and one nuclear regulatory factor [11] were also found to affect chemerin production (Figure 1). Indeed, the farnesoid X receptor (FXR) agonist GW4064 increased chemerin in HepG2 cells and primary hepatocytes, with this effect disappearing after FXR knockout [12]. Moreover, the RARRES2 promoter includes both a peroxisome proliferator-activated receptor γ (PPARγ)-binding sequence and a sterol regulatory element-binding protein 2 (SREBP2) binding site [13,14].

Abundant chemerin levels occur in the liver, adipose tissue, and placenta [15,16]. Yet, its mechanism of secretion is poorly understood, and changes in its gene expression do not necessarily parallel changes in its secretion [17,18]. This implies that chemerin secretion is subject to additional regulation [17,18]. Its synthesis starts with preprochemerin [8]. This precursor has a conserved consensus amino-terminal signal sequence and is thought to be sorted via conventional cellular secretory pathways [19]. Preprochemerin is secreted as chemerin163S or prochemerin, following cleavage of its 20 amino acid signal peptides. Prochemerin can be detected in circulation blood [20,21]. Proteolytic removal of the C-terminal helical segment by plasmin or angiotensin-converting enzyme type 2 results in the generation of both chemerin157S and chemerin156F from prochemerin [19,22,23]. Hepatic as well as whole-body knockdown of chemerin yielded an almost complete disappearance of circulating chemerin. This suggests that the liver is the predominant source of chemerin in blood [24]. Nevertheless, chemerin produced locally (e.g., in adipocytes and placenta) plays an important role in lipid metabolism and vascular function [25,26,27]. Chemerin was initially reported to induce chemoattraction and inflammation [8] in a calcium-dependent manner [8,28,29]. Yet, following its identification in adipocytes, it became gradually known as a novel adipokine affecting adipogenesis and lipid metabolism. This resulted in its association with obesity, diabetes, and metabolic syndrome [30,31,32]. Simultaneously, it was observed to affect vascular contraction, paving the way for its association with hypertension [25,26]. Adipokines facilitate the interaction between adipose tissue and other tissues [33]. The most extensively investigated adipokines are adiponectin and leptin. In general, during the transition from lean to obese, leptin levels increase, while adiponectin levels decrease [33], thereby decreasing the adiponectin/leptin ratio. Hence, increasing this ratio now emerges as a therapeutic goal. To what degree the adiponectin/chemerin ratio might be used to a similar extent is currently being debated [34].

### 2.2. Chemerin Receptors

Chemerin-like receptor 1 (CMKLR1), also known as chemokine receptor-like 1, ChemR23, or chemerin1 [35], was first reported in 1996. This receptor is predominately expressed in dendritic cells, monocytes, macrophages, endothelial cells, the placenta, lungs, muscle, heart, adipose tissues, skin and spleen [2,35,36]. CMKLR1 is the most widely investigated chemerin receptor. Chemerin binding to CMKLR1 results in G_i_ activation, which decreases cyclic adenosine monophosphate (cAMP), thereby resulting in the phosphorylation of extracellular signal-regulated kinase 1/2 (ERK1/2) and nuclear factor kappa B (NFκB) activation [37,38] (Figure 1). Interestingly, the dietary supplement resolvin E1, a bioactive oxygenated product of eicosapentaenoic acid (EPA), exerted potent anti-inflammatory effects in a CMKLR1-dependent manner [39]. This suggests that resolving E1 competes with chemerin for CMKLR1 binding, thus preventing its inflammatory effects. 

G protein-coupled receptor 1 (GPR1), also known as chemerin receptor 2 (chemerin2), was cloned in 1994 and identified as a chemerin receptor in 2008 [40,41]. It sequences homology with CMKLR1 is >40% [42]. Until today, as compared with CMKLR1, knowledge on GPR1 is limited. GPR1 occurs in the placenta, ovaries, testicles, skin, adipose tissue, skeletal muscle and brain [43,44]. GPR1 binds chemerin with high affinity, but this results in relatively weak biological signaling in a G_i_-dependent manner [40,45]. GPR1 may have more agonists than chemerin, for e.g., FAM19A1, a member of the family with sequence similarity 19 that was recently reported as a novel ligand for GPR1 in the brain [40,46]. 

CC motif chemokine receptor-like 2 (CCRL2) is believed to function as a chaperone protein, concentrating chemerin locally and thereby allowing optimal chemerin–CMKLR1 interaction [22,47]. It neither internalizes chemerin nor transduces signals [2,20]. CCRL2 is expressed in various tissues, including adipose tissue, breasts, the placenta, lungs, macrophages, dendritic cells, neutrophils and microglia [20]. 

## 3. Nutrients and Chemerin

**Figure 2:**
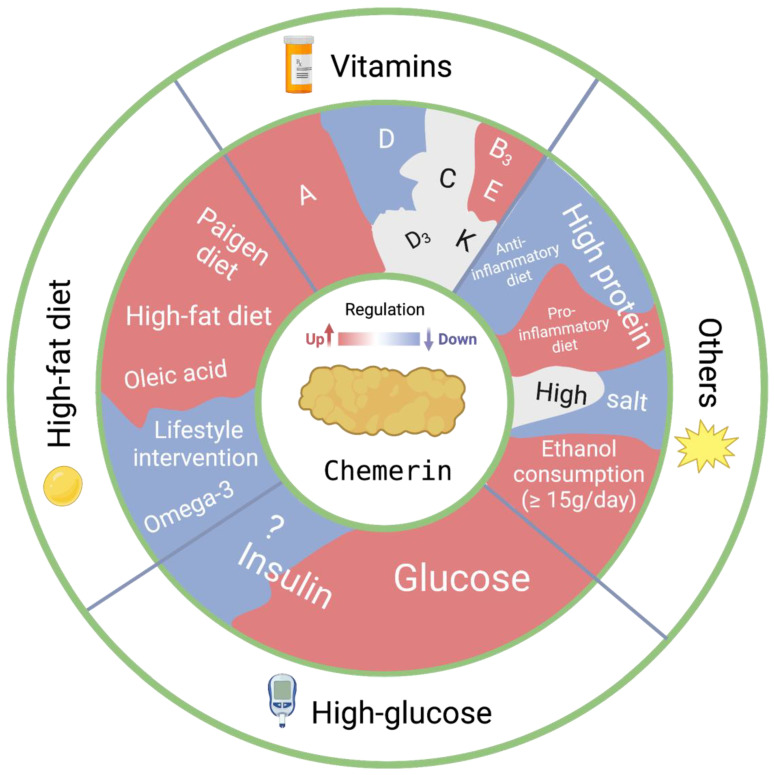
Effect of nutrient or diet on chemerin synthesis.

Nutrients and diet greatly affect chemerin production. Figure 2 summarizes the current knowledge.

### 3.1. Vitamins 

Vitamin A is derived from carotenoids and retinyl esters. This vitamin is essential, among others, for maintaining embryogenesis, vision, immune regulation and the metabolism of glucose and lipids [48]. Retinoids and retinoic acids are the primary metabolites of vitamin A, and some of their actions have been reported to involve chemerin [49]. This is not surprising given the fact that retinoic acid acts via the RAR, which directly induces the transcription of RARRES2, i.e., chemerin [50]. Indeed, incubation of intestinal cells, bone marrow stromal cells, endothelial cells and brown adipose tissue with retinoic acid upregulated chemerin [51,52,53]. Moreover, both beta-carotene and all-*trans* retinoic acid supplementation increased CMKLR1 expression in vivo as well as in vitro [54,55]. Endothelial CCRL2 expression also displayed retinoid acid-sensitive regulation in vitro [56]. No such findings have been reported for GPR1. 

Vitamin D supplementation led to improvement in rats with either pre-eclampsia or gestational diabetes mellitus, potentially because it lowered the elevated levels of chemerin in these models (see Section 4.3) [57,58]. While the protective effect of vitamin D on pre-eclampsia and gestational diabetes in humans is well established, to what degree this depends on chemerin lowering has not been investigated [59]. Additionally, both vitamin D-deficient obese children and type 2 diabetes mellitus patients display elevated chemerin levels [60,61], and circulating vitamin D levels negatively correlate with chemerin levels in breast cancer patients [62]. Yet, 1,25 dihydroxyvitamin D3, the active form of vitamin D, did not alter chemerin expression in renal tubular epithelial cells or endothelial cells [56,63]. One possibility is that the effects of vitamin D on chemerin are mediated via lipid lowering in vivo [64]. 

Vitamin C did not affect chemerin in adipocytes [65], and vitamin K absence in hepatocellular carcinoma patients did not alter chemerin [66]. Additionally, vitamin B3 increased chemerin mRNA levels in differentiated bovine preadipocytes [67], while vitamin E supplementation upregulated hepatic CMKLR1 mRNA expression [68]. 

### 3.2. High-Fat Diet and Glucose 

A high-fat diet, resulting in obesity and nonalcoholic fatty liver (NAFLD) in rats and mice, generally upregulates chemerin in blood, adipose tissue and liver [69,70,71]. Similarly, higher chemerin levels are observed at these same sites in obese and NAFLD patients in comparison with healthy humans [72,73,74]. Interestingly, an intensive lifestyle intervention consisting of dietary changes and resistance exercise programs over the course of several months lowers chemerin in obese subjects [75,76]. The fat-induced chemerin upregulation likely involves PPARγ, since the PPARγ agonist pioglitazone suppressed chemerin while the antagonist GW9662 did the opposite [77]. Remarkably, both chemerin knockout in vivo and chemerin knockdown in adipocytes decreased PPARγ expression, suggesting that chemerin–PPARγ interaction may occur in two directions [13,14]. Furthermore, in differentiated 3T3-L1 cells, SREBP2 knockdown prevented the oleic acid-induced rise in chemerin [13], confirming that this transcription factor contributes to chemerin synthesis. 

In mice, a high-fat diet upregulated CMKLR1 and CCRL2 in white adipose tissue and liver [78,79,80], while in rats chemerin knockout suppressed adipogenesis [81,82]. A high-fat diet also upregulated chemerin in pregnant mice, but decreased GPR1 [83]. Interestingly, GPR1 knockout mice exposed to a high-fat diet developed glucose intolerance with no change in body weight [84], while a lower body mass, body fat percentage and food intake was observed in CMKLR1 KO mice [85]. In apparent contrast with this latter finding, CMKLR1 and CCRL2 knockout mice exposed to a high-fat diet developed enhanced obesity [84,86], leading the authors to suggest that the net effect of the chemerin/CMKLR1 pathway might depend on the experimental setting.

A large cohort study has revealed a linear association between elevated levels of chemerin and the consumption of sugar-sweetened beverages [87]. Indeed, a high glucose challenge increased chemerin, both in 3T3-L1 cells and in mice in vivo, and this involved insulin [88]. Here, it is important to note that chemerin enhanced the insulin-stimulated glucose uptake in 3T3-L1 cells [89]. A similar chemerin upregulation, combined with increased CMKLR1 expression, was observed in human retinal pigment epithelium cells exposed to high glucose [90]. Yet, chemerin-mediated antagonism of insulin-induced signaling has also been observed, both in the vascular wall [91] and in human granulosa-lutein cells [92], although in the latter cells insulin still upregulated chemerin. Thus, while glucose upregulates chemerin in an insulin-dependent manner, chemerin may subsequently fine-tune the effects of insulin. Among others, this may involve the upregulation of pro-inflammatory cytokines via CMKLR1 [93], which will impair insulin signaling and promote insulin resistance [93]. In support of this concept, patients with proliferative diabetic retinopathy displayed higher serum chemerin and pro-inflammatory cytokine levels than patients with non-proliferative diabetic retinopathy [90].

Finally, omega-3 polyunsaturated fatty acids inhibit the secretion of chemerin from adipocytes [65,94]. This inhibition, which involved G-protein-coupled receptor 120, might contribute to the anti-inflammatory effects of omega-3 polyunsaturated fatty acids [95].

### 3.3. Protein, Salt and Alcohol

A healthy diet with a high protein and low carbohydrate content lowers chemerin, while the opposite occurs with a more pro-inflammatory (i.e., a low consumption of polyunsaturated and monounsaturated fats as well as fiber and high consumption of saturated fats) diet [96,97]. This was also true in patients with morbid obesity [98]. In contrast, a high intake of red meat, which associates with elevated levels of inflammatory markers, and a low intake of dairy, link to elevated chemerin levels [87].

Exposing Dahl salt-sensitive rats to a high-salt diet reduced circulating chemerin and increased its urinary secretion [99]. At the tissue level, high salt intake diminished chemerin particularly in adipocytes [100].

Chronic alcohol consumption upregulated chemerin in a dose-dependent manner, both in healthy humans (serum) [101,102] and in rats (serum and fat tissue) [101]. Chemerin mRNA levels were elevated in fat tissue in mice fed ethanol [103]. In patients with chronic pancreatitis, serum chemerin concentrations were higher in heavy drinkers compared with non-alcoholic patients [104]. The potential connection between alcohol, salt and chemerin levels may involve aldosterone. Notably, alcohol has been shown to increase aldosterone levels [105], whereas salt has been observed to decrease it [106]. Additionally, aldosterone has been found to elevate chemerin levels [107]. 

## 4. Potential Role of Chemerin in Metabolic and Cardiovascular Disease 

### 4.1. Lipid Metabolism

Chemerin not only stimulates adipogenesis but also facilitates lipid accumulation in a wide variety of cells [29,108,109,110,111,112]. In agreement with this concept, its levels and receptors are upregulated in differentiating preadipocytes. Moreover, obesity, NAFLD and nonalcoholic steatohepatitis (NASH) are all accompanied by elevated chemerin levels, while attenuating these conditions lowers chemerin [72,77,113,114]. Table 1 summarizes the genes that are currently believed to be involved in the effects of chemerin on lipid metabolism. Here, it should be noted that a methionine–choline-deficient (MCD) diet (a classical dietary model of NASH) has also been reported to decrease CMKLR1 [114,115] and chemerin in the liver [12]. These opposing effects on chemerin might relate to sex, as increased chemerin levels were observed in male animals exposed to a MCD diet [116], while MCD-fed females displayed chemerin lowering [12]. Moreover, in hepatocytes or matured adipocyte cells, the fatty acids EPA, docosahexaenoic acid, palmitate acid and oleic acid all induced lipid accumulation, while only the latter increased chemerin expression, with the former three decreasing this expression [12,94,114]. In an oral lipid tolerance test, chemerin decreased when switching from fasting to lipid uptake, reaching its lowest level after 4 h [117]. 

An obesogenic diet increases chemerin secretion from brown adipocytes, while cold stimulation caused the opposite [118,119]. Chemerin might contribute to temperature regulation, given that its overexpression decreased whole body and brown adipose tissue temperature in mice [120]. Chemerin overexpression additionally impaired metabolic homeostasis and induced glucose intolerance. These effects involved CMKLR1 and uncoupling protein 1. In addition, the chemerin–CMKLR1 axis is a physiological negative regulator of thermogenic beige fat, and targeting this pathway might be a novel strategy for obesity [121]. 

Circulating chemerin correlates positively with low-density lipoprotein (LDL) and negatively with high-density lipoprotein (HDL) [122,123]. Yet, the latter negative association particularly concerns large HDL, since a positive association was observed with both small and intermediate HDL. This suggests that chemerin is involved in the HDL maturing process [123,124]. LDL apheresis lowered circulating chemerin, implying that chemerin is bound, at least partly, to lipoproteins [125]. Future studies should investigate this possibility. 

### 4.2. Cardiovascular Effects

Chemerin levels are elevated in multiple cardiovascular diseases (Table 2) [126,127,128,129]. Chemerin is not only an independent risk factor for arterial stiffness [130], but in chronic kidney disease it also is a predictive marker of atherosclerosis [131,132]. This relates to the above-described effects of chemerin on the atherogenic process, involving vascular remodeling, lipid deposition and inflammation [93,133,134,135]. Indeed, the expression of chemerin and its receptor CMKLR1 in periaortic and pericoronary fat and foam cells determines atherosclerosis severity [136,137] and correlates with carotid plaque instability [138].

Recent data suggest that chemerin also exerts effects in cardiomyocytes, vascular smooth muscle cells, endothelial cells and fibroblasts, and might even originate from some of these cells. Tumor necrosis factor-α upregulated chemerin in murine cardiomyocytes, and in these cells chemerin induced apoptosis by activating caspase 9 and reducing protein kinase B (AKT) [139]. In rat cardiac fibroblasts, chemerin promoted cell migration by increasing reactive oxygen species (ROS), AKT and ERK1/2 [140]. Aldosterone induced chemerin synthesis in cardiac fibroblasts via Rho/ROCK/JNK signaling [141]. In endothelial cells, chemerin promoted angiogenesis and ROS production and decreased insulin signaling and nitric oxide production [2,91,142]. 

**Figure 3:**
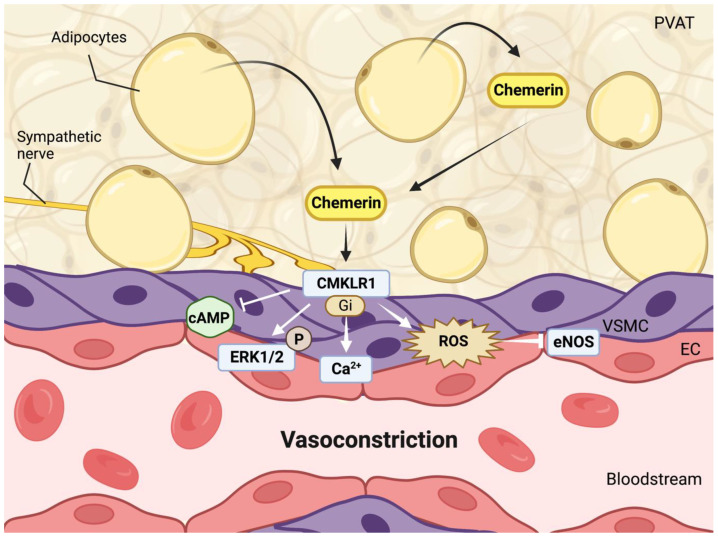
Chemerin-induced vasoconstriction involves both direct effects via its type 1 receptor (CMKLR1) on vascular smooth muscle cells (VSMCs), mediated via cyclic adenosine monophosphate (cAMP) reduction, upregulation of extracellular signal-regulated kinase 1/2 (ERK1/2) and reactive oxygen species (ROS), and indirect effects mediated via activation of the sympathetic nervous system. NO, generated by endothelial NO synthase (eNOS) in endothelial cells (EC), will counteract the effects of chemerin. Data are from references [24,26,28,35,143].

Vascular chemerin most likely originates from perivascular adipose tissue (PVAT), while CMKLR1 occurs in endothelial and vascular smooth muscle cells [26,143]. Exogenously added chemerin induced constriction via CMKLR1, G_i_ and calcium in isolated vessels (Figure 3), and this was enhanced after endothelial removal or during nitric oxide inhibition [26,28]. Without exogenous chemerin, endogenous chemerin derived from PVAT is also capable of inducing constriction, most likely by activating the sympathetic nervous system [143]. Remarkably, although both whole-body and hepatic chemerin knockdown abolished circulating chemerin [24], only whole-body knockdown also lowered blood pressure. This implies that chemerin from a non-hepatic source, most likely PVAT, contributes to blood pressure. To what degree the chemerin-induced upregulation of inflammatory cytokines in vascular smooth muscle cells [144] contributes to vessel contraction remains unknown.

### 4.3. Pregnancy-Related Problems

Chemerin is also a major player during pregnancy. Circulating chemerin levels normally fall in the first and second trimesters of pregnancy, and then increase during the third trimester, reaching the highest levels at late gestation, to fall again to pre-pregnancy levels shortly after delivery [145,146,147]. The placenta is a major contributor to this rise in circulating chemerin [110]. Since cord blood chemerin levels exceed those in maternal blood [146], maternal and fetal chemerin levels may act independently. Yet, maternal obesity is associated with higher cord blood chemerin levels [148,149]. How chemerin upregulation during pregnancy is regulated and whether chemerin affects the fetus are unknown. 

The high levels of chemerin in late pregnancy are suggestive of the possibility that they play a role in the preparation of delivery. This might require a delicate balance, given that overexpression of chemerin increases the risk of miscarriage [110]. Simultaneously, chemerin correlates positively with platelet count, which is relevant at the time of delivery to prevent hemorrhage [150,151,152]. Overall, excessively high maternal chemerin levels are indicative of a negative pregnancy outcome and a low birthweight, while cord blood chemerin levels associate positively with fetal birthweight [110,153,154]. In agreement with the former, intraperitoneal application of chemerin to pregnant mice with diabetes resulted in cognitive disorder in the offspring [155]. In the fetus, chemerin is expressed at the level of the intestine, where it peaks at 20–24 weeks of gestation to promote macrophage recruitment for gut development [52]. Thereafter intestinal chemerin expression returns to low levels. 

**Figure 4:**
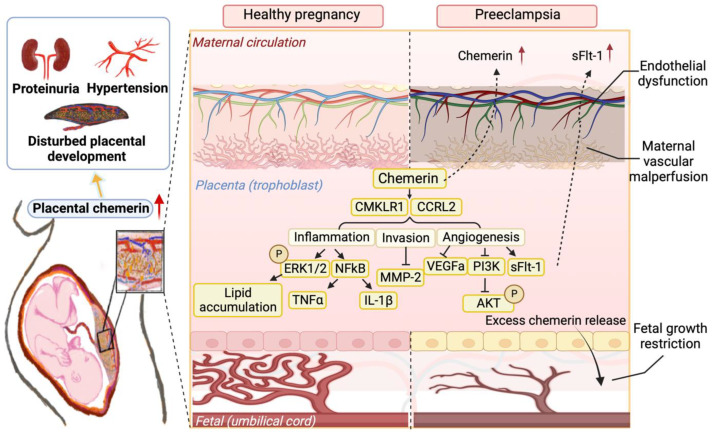
Placental trophoblast chemerin overexpression in mice induces a pre-eclampsia-like syndrome, involving hypertension and proteinuria, combined with diminished trophoblast invasion (by suppressing matrix metalloproteinase (MMP)-2), a disorganized labyrinth layer, and up-regulation of the anti-angiogenic factor soluble Fms-like tyrosine kinase-1 (sFlt-1) and the inflammation markers nuclear factor-κB (NFκB), tumor necrosis factor (TNF)-α and interleukin (IL)-1β, while downregulating vascular endothelial growth factor-a (VEGFa). The disturbed sFlt-1/VEGFa ratio involves the phosphoinositide 3-kinase (PI3K)/protein kinase B (AKT) pathway. Finally, extracellular signal-regulated kinase 1/2 (ERK1/2) upregulation might lead to lipid accumulation. Figure 4 summarizes the findings from references [110,158]. CMKLR1, chemerin-like receptor 1; CCRL2, CC motif chemokine receptor-like 2.

Serum chemerin is increased in pre-eclampsia, correlating with the severity of the disease and adverse neonatal outcomes [154,156]. In fact, its level in the first trimester may help to predict the occurrence of pre-eclampsia [157]. Importantly, the pre-eclamptic placenta releases more chemerin than a healthy placenta [110], supporting the concept that circulating chemerin in pregnancy is placenta-derived, and that the elevated chemerin levels in pre-eclamptic women originate in the placenta. Moreover, placental chemerin overexpression in mice induced a pre-eclampsia-like syndrome (Figure 4), characterized by high blood pressure, proteinuria, endothelial dysfunction and fetal growth restriction [110]. Placental chemerin overexpression simultaneously increased the circulating and placental levels of cholesterol, raising the possibility that chemerin might also contribute to dyslipidemia in pre-eclampsia [158]. A rat model of pre-eclampsia similarly displayed higher circulating chemerin levels [58]. In gestational diabetes mellitus (GDM), chemerin correlates with obesity and glucose homeostasis [50]. Yet, chemerin levels in the blood, adipose tissue and placenta are not necessarily elevated in GDM [159,160]—this may be limited to obese GDM women [161,162]. In such women, high cord blood chemerin levels were predictive for both maternal insulin resistance and large for gestational-age babies [148,149]. It is important to stress that adverse perinatal outcomes are linked to maternal cardiometabolic and neurocognitive outcomes [163,164]. This may represent the long-term consequences of inflammatory dysfunction, potentially involving chemerin.

### 4.4. Sex Differences

Sex hormones likely contribute to the synthesis and effects of chemerin. In humans, serum chemerin increases with age, and chemerin levels are higher in females than in males [117,165]. However, in type 2 diabetes and obesity cohorts, serum chemerin in males was higher than in females [166,167]. In the deoxycorticosterone acetate–salt rat model, chemerin deletion decreased blood pressure in females while increasing blood pressure in males [168]. Furthermore, chemerin levels in white adipose tissue were downregulated in female rats and upregulated in male rats after gonadectomy [169]. The latter coincides with observations in differentiated 3T3-L1 adipocytes, where testosterone decreased chemerin release into the supernatant. Yet in these cells estradiol was without effect [117], and in lean women with polycystic ovarian syndrome (PCOS), chemerin levels were upregulated versus obese PCOS women [170]. Chemerin was observed to suppress follicular steroidogenesis and may thus contribute to PCOS [170,171]. Additionally, chemerin levels were low in subfertile males, most likely due to their elevated luteinizing hormone levels [172], and this was suggested to reflect a link between chemerin and reproductive function.

**Table 2:** Circulating chemerin in various metabolic and cardiovascular diseases.

Country	Population	Number of Included Patients (n)		Chemerin Levels (ng/mL)		BMI		Age	Reference
		Control	Diseased	Control	Diseased	Control	Diseased		
USA	Obesity	10	37	76.2	147	<25	>25	54	[21]
Hungary	Obesity	50	50	405	590	<25	>25	43	[122]
Mauritius	T2D	142	114	249	250	≤25	>25	49	[31]
Saudi Arabia	T2D	38	41	89	99	>25	>25	44	[58]
Germany	T2D	29	29	191	219	>25	>25	56	[72]
USA	T2D	969	173	180	191	>25	>25	45	[162]
China	Atrial fibrillation	146	256	107.74	133.24	<25	<25	60	[126]
China	Coronary artery disease	191	239	45.7	48.7	≤25	≤25	62	[127]
China	Coronary artery disease	56	132	90	111	<25	<25	62	[128]
China	Coronary artery disease	50	50	133	189	≤25	≤25	60	[134]
Korea	Obesity and arterial stiffness	35	33	106	120	<25	>25	52	[129]
Canada	Stable and unstable carotid atherosclerotic plaque		165		208		>25	70	[138]
Austria	Hypertension *	A total of 495		155	180	>25		65	[131]
	T2D *			170	192				
	MetS *			163	201				
Netherlands	Pre-eclampsia	29	30	149	287	≤25	≤25	32	[110]
Germany	Pre-eclampsia	37	37	205	250	<25	<25	30	[145]
Turkey	Pre-eclampsia	46	88	200	358	>25	>25	27	[154]
China	Pre-eclampsia	477	41	181	312	<25	≤25	26	[157]
Germany	GDM	80	40	218	230	<25	<25	30	[160]

Abbreviations. T2D, type 2 diabetes; MetS, metabolic syndrome; ACE, angiotensin-converting enzyme; AT1, angiotensin II type 1; GDM, gestational diabetes mellitus. * these three populations are from one cohort.

## 5. Conclusions 

Chemerin is a novel player that might contribute to a wide variety of cardiovascular diseases, amongst others, by stimulating adipogenesis, inflammation and contraction, and by influencing thermogenesis, steroidogenesis and insulin signaling. Its concentrations vary widely, partly in a sex-dependent manner, and vitamin A, fat, glucose and alcohol generally upregulate it, while omega-3, salt and vitamin D suppress chemerin. Dietary measures rather than drugs acting as chemerin receptor antagonists might become novel tools to suppress chemerin effects, thereby potentially improving diseases such as atherosclerosis, diabetes, hypertension and pre-eclampsia. However, more detailed studies are required to fully understand chemerin regulation. 

## Figures and Tables

**Table 1:** Genes and proteins that are involved in the effect of chemerin on lipid metabolism.

Related Genes or Proteins	Disease or Model	Sample Type	Species	Reference
CMKLR1; IL6	NAFLD	Liver	Human	[72]
hsCRP	Obesity	Serum	Human	[73]
CMKLR1; PPARγ	T2D	Liver, gastrocnemius	Rat	[77]
ERK5; p-ERK5	Obesity	Osteoclast	Mouse	[78]
PI3K; AKT; p-AKT	Obesity	Kupffer cells	Mouse	[79]
insulin; CCRL2; AKT; p-AKT; ERK; p-ERK	Obesity	Visceral adipose tissue	Mouse	[80]
CMKLR1; ERK1; ERK2; PPARγ; adiponectin; perilipin; FASN; HSL; GLUT4; IR; TNFα; IL6; leptin; UCP1	Obesity; adipogenesis	Adipocytes (3T3-L1; brown adipose tissue)	Mouse	[81]
PPARγ; adiponectin; FAS; perilipin; leptin	Adipogenesis	Adipose tissue	Mouse	[82]
GPR1; GLUT3; AKT; p-AKT; PPARγ; FABP4	GDM; obesity	Placenta	Human; Mouse	[83]
Insulin; AKT; p-AKT	Insulin challenge	Adipocytes (3T3-L1; primary human adipocytes)	Human; Mouse	[86]
Insulin; AKT; p-AKT	T2D; obesity	Human vascular smooth muscle cells, mouse aortas	Human; Mouse	[89]
CMKLR1; insulin; IRS1; p-IRS1	T2D	Liver, adipose tissue	Mouse	[90]
HSL; LPL; leptin; PPARγ; CEBPα; FABP4	Adipogenesis	Bovine intramuscular adipocytes	Bovine	[108]
Cyclophilin D; UCP1; UCP2; PRDM16; PEPCK; DGAT-2; DIO-2	Obesity	Brown adipose tissue	Mouse	[109]
CMKLR1; TNFα; IL-1β; NFkB; PI3K; AKT; p-AKT	Pre-eclampsia	Placenta	Mouse	[110]
GPR1; SREBP1c; FASN; ACC1; DGAT-2; SCD-1; TNFα; IL6; SOCS3	NAFLD	Human hepatoma cell line HepG2	Human	[112]

Abbreviations. CMKLR1, chemerin-like receptor 1; IL6, interleukin 6; hsCRP, high-sensitivity C-reactive protein; PPARγ, peroxisome proliferator-activated receptor γ; ERK, extracellular signal-regulated kinase; p-ERK, phosphate extracellular signal-regulated kinase; PI3K, phosphoinositide 3-kinase; AKT, protein kinase B; p-AKT, phosphate protein kinase B; CCRL2, CC motif chemokine receptor-like 2; FASN, fatty acid synthase; HSL, hormone-sensitive lipase; GLUT4, glucose transporter type 4; GLUT3, glucose transporter type 3; IR, insulin receptor; TNFα, tumor necrosis factor alpha; UCP, uncoupling protein; GPR1, chemerin type 2 receptor; FABP4, fatty acid-binding protein 4; IRS1, insulin receptor substrate-1; p-IRS1, phosphate insulin receptor substrate-1; LPL, lipoprotein lipase; CEBPα, enhancer-binding protein alpha; PRDM16, positive regulatory domain zinc finger region protein 16; PEPCK, phosphoenolpyruvate carboxykinases; DGAT-2, diacylglycerol O-acyltransferase 2; DIO-2, type II iodothyronine deiodinase; NFκB, nuclear factor-κB; SREBP1c, sterol regulatory element-binding protein 1; ACC1, acetyl-CoA carboxylase 1; SCD-1, stearoyl-CoA-desaturase 1; SOCS3, suppressor of Cytokine Signaling-3; T2D, type 2 diabetes; GDM, gestational diabetes mellitus.

## Data Availability

Not applicable.

## Supplementary Materials

The following supporting information can be downloaded at: https://www.mdpi.com/article/10.3390/nu15132878/s1, Figure S1: Flowchart of study selection.
