# Supplementary material for: The Role of Chemerin in Metabolic and Cardiovascular Disease: A Literature Review of Its Physiology and Pathology from a Nutritional Perspective
Source: Nutrients. 2023 Jun 25;15(13):2878. doi: 10.3390/nu15132878 (PMC10343651; doi:10.3390/nu15132878)
Supplement: Supplementary file 1 [file nutrients-15-02878-s001.zip › nutrients-2426012-supplementary.pdf]

Search strategy and study selection

A comprehensive systematic literature search was performed on 15th April 2023, utilizing PubMed and Google Scholar as the search database. The search terms employed were as follows: 1) "chemerin" AND "nutrient" OR "diet", 2) "chemerin" AND "vitamin", 3) "chemerin" AND "high-fat", 4) "chemerin" AND "high sugar" OR "high glucose", 5) "chemerin" AND "high protein", 6) "chemerin" AND "high salt", 7) "chemerin" AND "alcohol" OR "ethanol consumption". After the removal of duplicate entries, the full texts were evaluated based on pre-defined inclusion and exclusion criteria.

Inclusion and exclusion criteria

In our analysis, we included every study that that have reported the impact of nutrient, diet, or compound supplementation on the mRNA or protein expression levels of chemerin in both *in vitro* and *in vivo* studies. Review articles, editorials, correspondence, case reports, case series, and articles published in languages other than English have been excluded from the analysis.

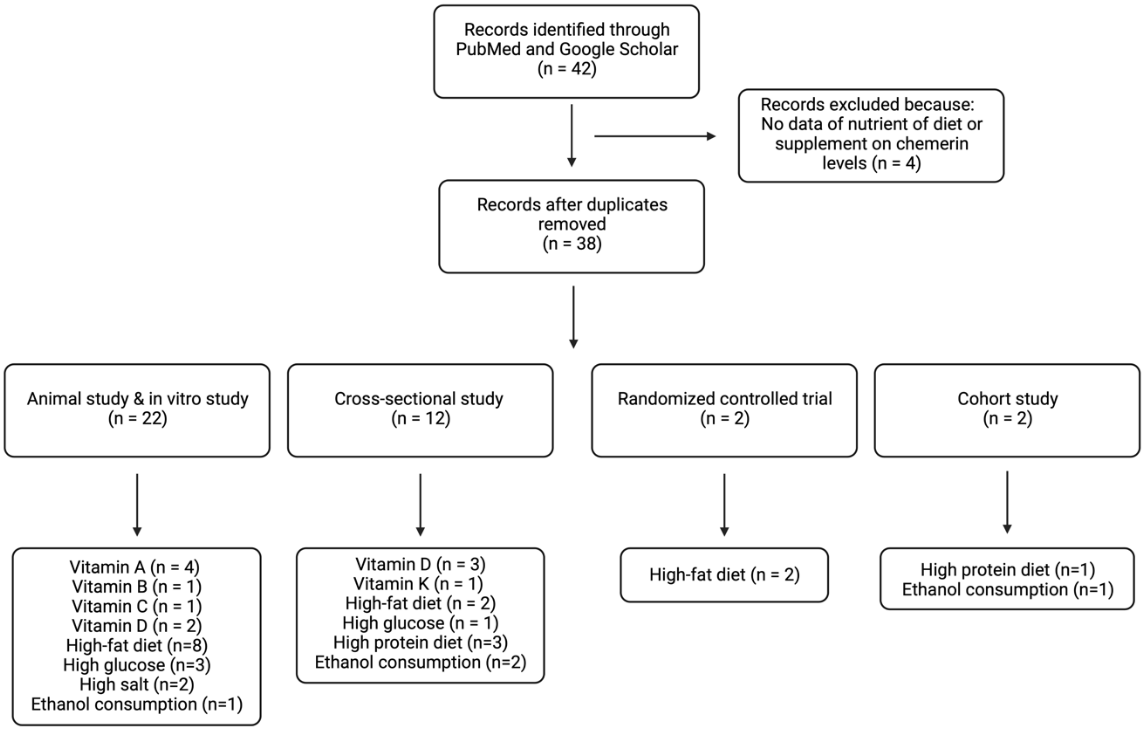

Figure S1. Flowchart of study selection.
